# SARS-CoV-2 variants resistant to monoclonal antibodies in immunocompromised patients constitute a public health concern

**DOI:** 10.1172/JCI168603

**Published:** 2023-03-15

**Authors:** Arturo Casadevall, Daniele Focosi

**Affiliations:** 1 Department of Molecular Microbiology and Immunology, Johns Hopkins Bloomberg School of Public Health, Baltimore, Maryland, USA.; 2 North-Western Tuscany Blood Bank, Pisa University Hospital, Pisa, Italy.

## Abstract

COVID-19 in immunocompromised hosts has emerged as a difficult therapeutic management problem. Immunocompromised hosts mount weak responses to SARS-CoV-2 and manifest infection outcomes ranging from severe disease to persistent infection. Weakened immune systems mean greater viral loads and increased opportunities for viral evolution. Gupta, Konnova, et al. report the emergence of resistant SARS-CoV-2 variants in immunocompromised patients after monoclonal antibody (mAb) therapy. mAbs target only a single determinant in the viral Spike protein, which is a weakness of such therapy when treating a mutagenic and variable virus. Hence, the emergence of mAb resistance could have been anticipated, but its documentation is important because it has major public health implications, since such resistant variants have the potential to spread and escape vaccine immunity. For immunocompromised patients, these findings suggest the need for combination therapy with antiviral drugs and the use of polyclonal antibody preparations such as convalescent plasma.

## Treatment-resistant variants

In this issue of the *JCI*, Gupta et al. (1) report the systematic emergence of SARS-CoV-2 variants expressing Spike mutations in immunocompromised patients treated with different anti-Spike monoclonal antibodies (mAbs) across different pandemic waves. Antibody-based antiviral therapies largely work by neutralizing virions. Resistance to neutralization can be either basal or develop after treatment. Gupta et al. (1) show that the majority of such mutations were treatment specific and conferred specific treatment resistance. These findings closely parallel and confirm previous case reports and small case series reporting that mAb therapy, such as sotrovimab (2), can select for treatment-resistant variants in immunosuppressed patients. Although the immediate clinical importance of the findings by Gupta et al. (1) has been dulled by the withdrawal of all anti-Spike mAb therapies due to the emergence of resistant Omicron sublineages, their experience has valuable implications for other and future antiviral mAbs. mAb treatment–emergent resistance in immunocompromised patients could have been anticipated given what we know from the history of antibody-based therapies, the mechanism of action of mAbs, and the mutation-prone nature of SARS-CoV-2. The evidence that this resistance has occurred with notable incidence has tremendous implications for future therapy and public health policies in response to the pandemic.

**Figure 1:**
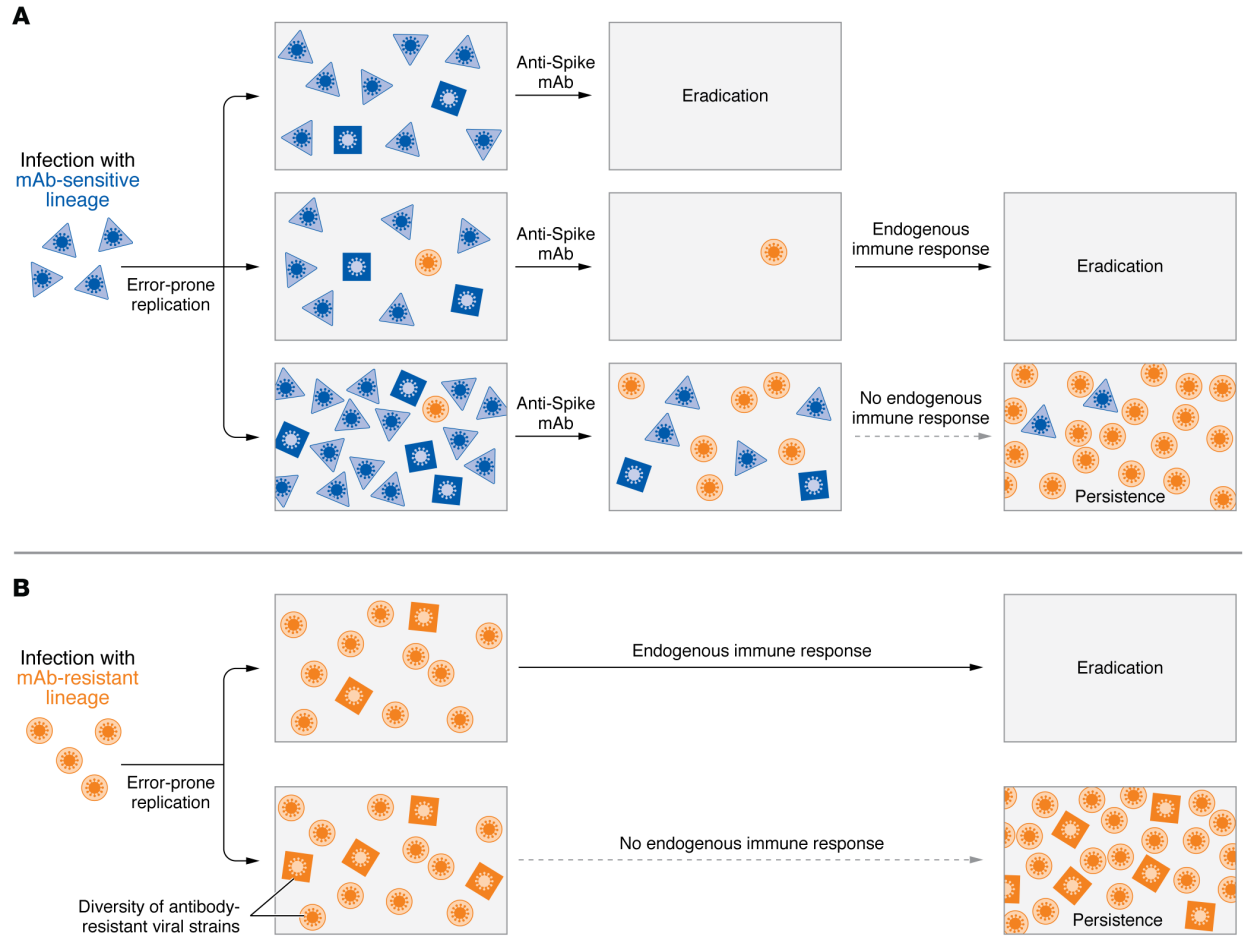
mAb therapies select for escape variants in SARS-CoV-2 infection. (**A**) SARS-CoV-2 infection with susceptible viral strains (blue squares and diamonds) has several potential outcomes after mAb therapy, including the emergence of antibody-resistant strains (orange circles). (**B**) Infection with a mAb-resistant virus (orange circles) can further diversify viral strains (orange squares and diamonds), yielding strains with the potential for evading vaccine immunity and initiating additional infection waves within the population.

Since many viral infections, including SARS-CoV-2, produce a quasi-species swarm in vivo because of the error-prone viral replication, there is always the concern that therapies will select for escape variants. Each mAb recognizes a single epitope on viral surface antigenic determinants, and immunocompromised patients typically have higher baseline viral loads and delayed viral clearance (as the authors demonstrate for SARS-CoV-2). Such a conundrum creates an ideal landscape for treatment-emergent resistance (Figure 1).

It is relatively easy to select for antibody escape variants in vitro by incubating specific mAbs with replicating virus, and this process has been carried out for Chikungunya virus (3), respiratory syncytial virus (RSV) (4), and SARS-CoV (5). Escape variants can emerge during mAb therapy of RSV experimental infection in Cotton rats (6), or through prophylactic mAb pre-exposure in humans (7). Hence, the findings of Gupta et al. (1) are not surprising, but they are of great importance given the implications for immunocompromised patients with COVID-19 and for potential directions of the pandemic.

## Challenges in immunocompromised patients

As we enter the fourth year of the COVID-19 pandemic, the problems associated with SARS-CoV-2 infection in immunosuppressed hosts are emerging as perhaps the most pressing therapeutic problem and the greatest challenge for the control of the virus. COVID-19 in immunocompromised patients present three major challenges: (a) these patients do not respond well to vaccination, reducing the options for preventing severe disease; (b) disease can be severe and infection often becomes persistent, which can interfere with eligibility for further therapies for underlying conditions such as cancer; and (c) as reported by Gupta et al. (1), immunocompromised patients harbor higher baseline viral load that can facilitate emergence of mAb resistance.

The challenge of COVID-19 in immunocompromised patients presents physicians with a difficult therapeutic option, especially when the infection becomes persistent. These individuals can require multiple rounds of therapy. Unlike immunocompetent patients who mount strong and effective responses to vaccination, these individuals have weak immune responses and, consequently, therapies such as small molecule antivirals do not benefit from concomitant immunity. However, all monotherapies, whether these involve mAbs or small molecule antivirals, run the risk of selecting for resistant viral variants that will abrogate its effectiveness.

The emergence of mAb-resistant variants in immunocompromised patients is also a potential public health threat since these variants could be fit enough to be transmitted to family members (8) and health care workers. Such mAb-resistant variants could also theoretically manifest increased resistance to vaccine-induced immunity because of major Spike protein changes. In fact, there has been concern that the Omicron variant may have originated from a chronic infection in an immunosuppressed patient (9).

## Benefiting patients while minimizing public health risks

At the time of this writing, all previously available mAb therapies have failed due to the emergence of antibody-resistant variants. There are currently efforts to develop additional therapeutic mAbs, but these too could prove vulnerable to emerging variants unless the development of resistance is mitigated. One possibility for reducing the likelihood that a single variant expresses mutations that enable escape from available mAbs is to use mAb cocktails that target the virus at different epitopes. Proof of principle for this strategy in effectively reducing the emergence of escape mutants was shown with H5N1 influenza virus in mice that received combinations of mAbs (10). However, the emergence of resistant viruses from cocktails composed of two therapies has already been demonstrated (11–14), and more resistant variants are likely to arise when one of the cocktail components is ineffective at baseline (15, 16). Maybe cocktails of three, as developed, e.g., for Ebolavirus (17), could be more successful, but cost remains an obvious issue.

The loss of mAbs for prevention and therapy of COVID-19 is tragic given that these treatments were beneficial to immunocompromised patients and their absence leaves physicians with fewer options when confronting SARS-CoV-2 infections. In fact, many frail patients have baseline contraindications to Paxlovid, and the short schedules authorized for both Paxlovid and remdesivir may not be adequate to clear the virus. Fortunately, we have an alternative that is immediately available in the form of COVID-19 convalescent plasma (CCP), which has shown mortality benefits in the immunocompromised population in the pre-Omicron era (18–20). Importantly, CCP collected from vaccinees preserves in vitro efficacy against all recent Omicron sublineages (21, 22). And, given the high prevalence of infection in vaccinated populations, such CCP is currently in plentiful supply. In contrast to mAbs, CCP is a polyclonal product that includes antibodies of different specificities and effector functions and is thus less likely to select for antibody-resistant variants. In fact, CCP has been used in rescue therapy of immunocompromised patients who developed antibody-resistant variants during mAb therapy (23, 24). One important caveat when considering antibody therapies for COVID-19 in immunocompromised individuals is that this patient group is heterogeneous and we need to have a better understanding of which patients are more likely to benefit (e.g., seronegatives). The COVID-19 pandemic has shown that antibody therapies can be effective while also reminding us that these are more complex than small molecule antiviral drugs. Going forward, we need additional high-quality studies to learn how to use antibody-based therapies optimally such that we can benefit individual patients while minimizing the public health risks to society from the emergence of immunity-evading SARS-CoV-2 variants.

## Version 1 03/15/2023

Electronic publication

## Version 2 03/16/2023

Article title updated
